# The Constellation of Macrovascular Risk Factors in Early Onset T2DM: A Cross-Sectional Study in Xinjiang Province, China

**DOI:** 10.1155/2018/3089317

**Published:** 2018-04-03

**Authors:** Mingchen Zhang, Jiangfeng Mao, Ablikm Tuerdi, Xiaoyun Zeng, Li Quan, Shan Xiao, Jun Zhu, Hua Yao

**Affiliations:** ^1^Department of Endocrinology, First Affiliated Hospital of Xinjiang Medical University, Urumqi, China; ^2^Postdoctoral Research Station of Public Health, School of Public Health, Xinjiang Medical University, Urumqi, China; ^3^Department of Endocrinology, Peking Union Medical College Hospital, Key Laboratory of Endocrinology, Ministry of Health, Beijing, China; ^4^The Key Laboratory of Xinjiang Metabolic Disease, Clinical Medical Research Institute, First Affiliated Hospital of Xinjiang Medical University, Urumqi, China

## Abstract

**Background:**

Despite a rapid popular of early onset type 2 diabetes (defined as diagnosis at <40 years old) recently, there is a lack of studies on this population in economically undeveloped area. We aimed to investigate the risk factors of macrovascular complications in the early onset T2DM patients in Xinjiang, China.

**Methods:**

A cross-sectional survey of 1736 consecutive patients with T2DM was conducted. Macrovascular complications and risk factors were documented. Another nondiabetic population matched with age and sex was as a control group. Logistic regression analysis was performed to obtain odds ratios (OR) for macrovascular complications in early and late onset T2DM, respectively.

**Results:**

The final analysis consisted of 1036 late onset and 219 early onset T2DM patients. The mean HbA_1c_ in the early onset group was higher than that in the late onset group (9.1 ± 2.4% versus 8.3 ± 2.2%, *P=*0.039) despite a higher proportion of patients in the early onset group receiving insulin treatment (73.1% versus 58.7%, *P* < 0.001). Compared to the control, early onset patients had higher blood pressure and worse lipid profiles (all *P* < 0.01). More than half of the early onset T2DM patients already had macro- and microvascular complications, despite of their young age (39.5 ± 10.8) and short DM duration (6.6 ± 8.0). In the early onset group, women had a ~3-fold hazard of atherosclerotic plaques compared with men (OR 3.22, 95% CI 1.53–6.78).

**Conclusions:**

Patients with early onset T2DM have worse glycemic control and higher burden of atherogenic risk factors. The prevalence of macro- and microvascular complications is astonishingly high in these young adults with T2DM. Moreover, young women with T2DM are more susceptible to cardiovascular complications than their male counterpart.

## 1. Introduction

Diabetes is one of the leading causes of death and disability. The number of patients with diabetes worldwide is projected to double over the next 30 years, mainly in a developing country; 85% of patients are categorized as having type 2 diabetes (T2DM), with associated mortality predominantly from cardiovascular diseases (CVDs) [1]. The phenotypic landscape of T2DM has evolved, with increasing numbers of people younger than 40 years being affected. Many Western countries have witnessed an increase in the prevalence of early onset T2DM [2, 3]. The impact of early onset T2DM is extensive. Recent evidence suggests that early onset T2DM is a more aggressive disease phenotype than the late onset cohort, and a premature development of micro- and macrovascular complications is the main concern for these young patients [4, 5]. Because early onset T2DM is a relatively new phenomenon, having only been recognized in the past few decades, the data of clinical outcomes are limited.

During the past three decades, the prevalence of diabetes in China increased from 1.3% in 1986 to 11.6% in 2010; 5.7% of these people were younger than 40 years at diagnosis in 2010 [6, 7]. Xinjiang Province is located in the northwest of China and composed of more than 13 ethnic groups. The prevalence of T2DM in Xinjiang Province has increased considerably over recent decades to an estimated 8.2% in urban areas and 6.1% in rural areas (approximately 2 million DM patients) [8]. There are few studies on the prevalence and risk factors of diabetes-related complications in early onset T2DM Han nationality population residing in economically developed areas of China [6, 9]. However, no study focused on early onset diabetes in economically underdeveloped and multiethnic areas such as Xinjiang. To address this gap in knowledge, we conducted a cross-sectional study which aimed to estimate the phenomenon of early onset T2DM patients residing in Xinjiang, China.

## 2. Materials and Methods

### 2.1. Study Population

This cross-sectional and noninterventional study was conducted from May 2014 to September 2015. A total of 1736 consecutive T2DM patients, who underwent diabetic complication evaluation in the outpatient clinic, the First Affiliated Hospital of Xinjiang Medical University, were screened. 349 individuals were excluded for incomplete diabetic complication tests, 87 individuals for incomplete laboratory tests, and 45 individuals for incomplete physical exam. The final analysis consisted of 1255 patients who had met the inclusion and exclusion criteria. The inclusion criteria included all the following points: (1) T2DM diagnosed based on the 1999 World Health Organization's definition [10]; (2) age 18 years or older; (3) and fulfilled comprehensive physical examination, laboratory tests, and complication evaluation tests. Exclusion criteria were as follows: (1) patients with type 1 diabetes mellitus, other specific types of diabetes mellitus, and unclear types of diabetes; (2) pregnancy or breast feeding; (3) being unable to complete the survey owing to mental illness or other reasons; (4) recent history of acute illness; and (5) drug or alcohol abuse. The patient's gender, age, weight, height, smoking status, date of diabetes diagnosis, and family history of DM were recorded. Past medical history and medications for T2DM were documented. Evaluations comprised detailed medical history, physical examination, laboratory measurements, and diabetic complication studies. The control group, including 478 subjects after matching for age and gender with early onset DM, is derived from another epidemiological study, which is aiming to investigate the prevalence of metabolic syndrome in Urumqi community, Xinjiang Province. Each of them had normal plasma glucose level after OGTT.

### 2.2. Anthropometric and Laboratory Measurements

Height and weight were measured to the nearest 0.1 cm and 0.1 kg, respectively. Body mass index (BMI) was defined as weight (kg) divided by height (m) squared. Blood pressure was measured after the subject had rested for at least 10 min in a sitting position. Blood samples were collected in the morning at fasting state. Laboratory data on fasting glucose, HbA_1c_, lipid profiles, and diabetic complication evaluation were recorded. Biochemical parameters such as plasma glucose and lipids (triglycerides, total cholesterol, HDL cholesterol, and LDL cholesterol) were measured by an autoanalyzer (ADVIA1650; Siemens, NY, USA) with commercially available kits. HbA_1c_ was measured by high-performance liquid chromatography using the VARIANT II Hemoglobin Testing System (Bio-Rad Laboratories). Urinary albumin was analyzed with an enzyme-linked immunosorbent assay.

### 2.3. Definition

In consistent with the previous studies, early onset T2DM in this study was defined as T2DM diagnosed younger than 40 years [3, 6, 9]. Type 1 diabetes mellitus was defined as patients with a history of ketoacidosis or GAD antibody positive when they were diagnosed with diabetes mellitus, with a large amount of ketonuria, and required persistent insulin therapy within the 1st year after the diagnosis. Hypertension was defined as systolic blood pressure (SBP) ≥ 140 mmHg or diastolic blood pressure (DBP) ≥ 90 mmHg or treatment with antihypertensive medications. Microalbuminuria was defined as urinary albumin ≥ 30 mg/24 h after excluding other causes of kidney damage, urinary tract infection, and hematuria [11]. The diagnosis of diabetic retinopathy was made by ophthalmologist(s) based on typical funduscopic findings of diabetes, including background, preproliferative, proliferative, and maculopathy. The diagnosis of diabetic neuropathy was made based on electromyography (EMG). EMG was considered to be positive when it demonstrated distal symmetric polyneuropathy and other causes of neuropathy were excluded. Carotid atherosclerosis was determined by B-mode ultrasound (ATL HDI 3000-CV; Phillips Medical Systems). This procedure was performed by an experienced ultrasonographer while the patient was supine with the neck extended in a slightly lateral rotation. Average carotid intima media thickness was calculated, and a *B*-score was formed as follows: 0 = no alteration, 1 = wall thickness < 1 mm, 2 = plaque 1 to 2 mm, 3 = plaque 2 to 3 mm, 4 = plaque > 3 mm, and 5 = total occlusion of the lumen. A single *B*-score > 2 on any measurement was defined as carotid atherosclerotic plaque. All the evaluators were blinded to the other test results.

### 2.4. Statistical Analysis

We conducted all statistical analyses using the IBM SPSS version 21.0 (IBM Corp., Armonk, NY). Data were given as means ± SD for continuous variables or percentages (%) for categorical variables. The distribution of continuous variables was checked by a normality test. If normal distributions were not rejected, continuous variables were compared by Student's *t*-test. Otherwise, the comparison was done by Kruskal-Wallis test. Categorical variables were compared using chi-square test. Logistic regression analysis was used to obtain odds ratios (OR) and their 95% confidence intervals (CI) for macrovascular complications in the early and late onset T2DM groups, respectively. All the statistical tests are two-sided, and a *P* value < 0.05 was considered statistically significant.

### 2.5. Ethical Consideration

The study was designed to comply with the Declaration of Helsinki. Written informed consent was obtained from all participants, and the study was approved by the ethics committee of the First Affiliated Hospital of Xinjiang Medical University, China.

## 3. Results

### 3.1. Population Characteristics

Among the 1736 enrolled patients, 481 with key variables missing were excluded, and the remaining 1255 (219 early onset and 1036 late onset) were used in the analysis. These patients had a mean age of 55.5 years (SD 10.2), with 7.7 years (SD 6.7) history of diabetes. Their mean HbA_1c_, fasting plasma glucose, and 2-h postprandial glucose were 8.5% (SD 2.3), 9.9 mmol/L (SD 4.5), and 14.4 mmol/L (SD 5.4), respectively.

Clinical and biochemical data on patients with early and late onset diabetes are summarized in Table 1. A total of 219 (17.5%) were diagnosed with diabetes before 40 years of age, and the mean age of diagnosis was 34.3 years (SD 7.6).

The mean HbA_1c_ of adults with early onset was higher than that with late onset (9.1 ± 2.4% versus 8.3 ± 2.2%, *P* = 0.039), despite a higher proportion of patients in the early onset group receiving insulin treatment (73.1% versus 58.7%, *P* < 0.001). Compared to the late onset group, patients in the early onset group were more likely to be younger, to have a higher prevalence of diabetic family history, to be a smoker, to have a shorter duration of T2DM, and to have a lower BMI. Although patients in the early onset group were markedly younger than patients in the late onset group, the prevalence of hypertension and dyslipidemia was similar between the two groups, reflecting metabolic abnormalities occurred earlier in this group.

Since many metabolic parameters are highly correlated with age and gender, a total of 478 subjects with normal plasma glucose levels were selected as the control group after matching for age and gender with the early onset group. Compared to the control subjects, patients with early onset T2DM had a much higher incidence of hypertension (56.2% versus 33.9%, *P* < 0.001). Systolic and diastolic blood pressure, triglyceride concentrations, total cholesterol, and LDL cholesterol levels in the early onset group were increased, while HDL cholesterol level was decreased compared with the control group (Table 1).

### 3.2. The Prevalence of Diabetic Complications

**Figure 1:**
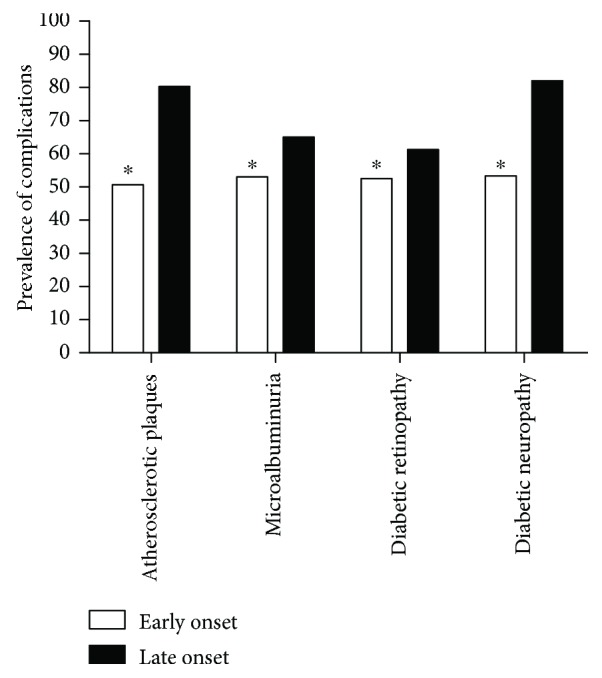
The prevalence of diabetic complications in early versus late onset T2DM. ^∗^All *P* values for chi-square test < 0.01.

Early onset diabetes may be associated with the development of complications at an early stage of life; therefore, we compared the prevalence of diabetic complications between the early and late onset groups. Patients in the late onset group had a much higher incidence of atherosclerotic plaques, diabetic retinopathy (DR), diabetic neuropathy, and microalbuminuria compared with the early onset group (late onset versus early onset: 80.3% versus 50.7% for atherosclerotic plaques; 61.3% versus 52.5% for DR; 82.0% versus 53.4% for diabetic neuropathy; 65.0% versus 53.0% for microalbuminuria; all *P* < 0.01) (Figure 1). (The detailed information about macrovascular complications is shown in Supplementary Table 1.)

### 3.3. The Management of Traditional Cardiovascular Risk Factors

**Figure 2:**
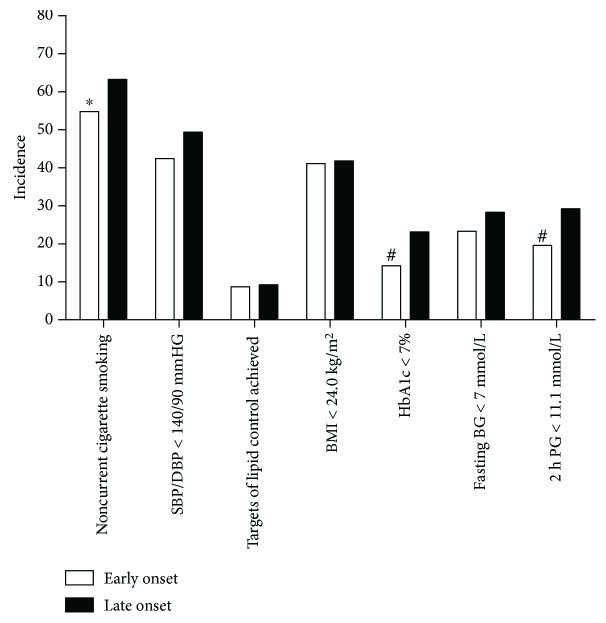
Control of traditional cardiovascular risk factors in patients with early and late onset T2DM. *y*-axis: incidence of achieved control targets of traditional cardiovascular risk factors. Targets of lipid control were defined as LDL cholesterol less than 2.6 mmol/L, HDL cholesterol greater than 1.0 mmol/L in men and 1.3 mmol/L in women, and triglyceride less than 1.7 mmol/L whether patients were on lipid-lowering drugs or not. BG, blood glucose; PG, postprandial glucose. ^∗^ *P* < 0.05, ^#^ *P* < 0.01; all *P* values for chi-square test.

Both groups had poor management on traditional cardiovascular risk factors (Figure 2): in either group, less than half achieved blood pressure targets and less than 10% achieved lipid targets. In the early onset T2DM group, smaller portion of patients achieved the target of glucose control (14.2% versus 23.1% for HbA_1c_ < 7%; 19.6% versus 29.2% for 2-h postprandial glucose < 11.1 mmol/L; all *P* < 0.01). They were more likely to be smokers (45.2% versus 36.8%, *P* < 0.05). (The detailed information about antihypertensive and statin therapies is shown in Supplementary Table 2.)

### 3.4. The Independent Risk Factors on Atherosclerotic Plaques

Multiple logistic regression analysis was done to assess the independent risk factors on atherosclerotic plaques in early and late onset T2DM, respectively. The variables in the multivariate model included age, gender, age of diabetes diagnosis, duration of diabetes, BMI, systolic and diastolic blood pressure, hypertension, smoking, fasting glucose, 2-h postprandial glucose, HbA_1c_, total cholesterol, LDL cholesterol, HDL cholesterol, and triglyceride. In the early onset group, females had a ~3-fold hazard of atherosclerotic plaques compared with male adults (OR 3.22, 95% CI 1.53–6.78). Other risk factors included age (OR 1.05, 95% CI 1.02–1.08), SBP (OR 1.04, 95% CI 1.02–1.07), and 2-h postprandial glucose (OR 1.01, 95% CI 1.00–1.03). The results are presented in Table 2. By contrast, in the late onset group, females had a much lower risk of atherosclerotic plaques relative to male adults (OR 0.25, 95% CI 0.17–0.37). Other risk factors associated with atherosclerotic plaques in the late onset group included age (OR 1.05, 95% CI 1.03–1.07), DBP (OR 1.01, 95% CI 1.00–1.03), 2-h postprandial glucose (OR 1.04, 95% CI 1.01–1.07), and LDL cholesterol (OR 1.18, 95% CI 1.01–1.38). The results are presented in Table 3.

## 4. Discussion

There is a marked increase in the prevalence of diabetes in China with more and more young people affected by the disease [12]. Our study found that (1) early onset T2DM accounted for 17.5% of the diabetes outpatient population in our tertiary care center in Xinjiang, China. Poorer glycemic control (compared to the late onset group) and higher burden of atherogenic risk factors (compared to the control group) were documented in the early onset cohort; (2) the incidence of micro- and macrovascular complications was astonishingly high (up to ~50%) in these young adults with T2DM; (3) risk factors for macrovascular complications were different in the early and late onset groups, and women were more susceptible to cardiovascular complications in the early onset group.

Although T2DM is traditionally considered as a disease mainly affecting the elderly, the age at diagnosis has gradually reduced in recent years. A rapid increase in the incidence of early onset T2DM has become a new trend all over the world. From 1990 to 2000, the number of patients with early onset T2DM increased 9 times in New York [13]. Recent large survey in China showed that 5.7% and 44% of adults aged below 40 years have diabetes or prediabetes, respectively [14]. Consistent with the previous studies, our data found early onset T2DM accounted for 17.5% of the diabetes outpatient population in Xinjiang, China. These data suggest that adults with early onset T2DM represent a substantial proportion of patients consuming medical resource even in economically underdeveloped areas of China. Of note, patients with early onset T2DM, despite of younger ages and shorter durations of diabetes, had poorer plasma glucose control than those patients with late onset [8, 15, 16]. Thus, prevention of diabetes, especially among individuals aged below 40 years, should be regarded as a public health emergency in such area.

Diabetic nephropathy (DN) and diabetic retinopathy (DR) are major microvascular complications of T2DM [17]. Previous reports showed that microalbuminuria was present in ~25% of T2DM patients when diagnosis was made. The incidence would increase to 28–42% and 60% at 5 and 10 years after the diagnosis was made [18]. Beyond our expectation, the incidence of microalbuminuria was 53.0% in our early onset patients, despite of their short disease duration and young age. The reported rates of DR in early onset T2DM population were 4–40%, varied considerably depending on the different study methods [18–20]. The incidence of DR in our early onset population was 53.4%, higher than previous reported [18–20]. In a UK study, 40% patients aged 13–35 years had evidence of neuropathy, and among them, 20% had evidence of skin ulceration [21]. In consistent, our study found that 53.4% of early onset T2DM patients had neuropathy. Our findings revealed that microvascular complications may occur early in the natural history of T2DM in young adults. Therefore, screening for microvascular complications is indispensable and should be initiated when young adult is diagnosed. Since it is conclusively established that the microvascular complications are directly related to glycemic [22] and blood pressure control [23, 24], more intensive control on hyperglycemia and hypertension is required in these young patients with T2DM.

Macrovascular complications are the primary cause of mortality, with myocardial infarction (MI) and stroke accounting for 80% of all death in T2DM patients [25]. According to the United Kingdom Prospective Diabetes Study (UKPDS), the main risk factors for cardiovascular disease in diabetes are raised LDL cholesterol and HbA_1c_, lower HDL cholesterol, higher systolic blood pressure, and current smoking [26]. Our data documented that early onset T2DM patients had a more adverse cardiovascular risk profile compared with age- and sex-matched controls. Hillier and Pedula demonstrated that the hazard of MI in early onset T2DM patients was 14-fold higher than in control subjects (HR 14.0, 95% CI 6.2–31.4) [4]. These data suggested that young patients with T2DM may lose the protection of youth. Previous studies demonstrated that early onset cohorts have similar or more adverse risk factors for cardiovascular diseases compared to late onset population [27]. Consistently, we observed that a high proportion had hypertension (51.1%), morbid obesity (58.9%), and dyslipidemia (91.3%) in early onset T2DM cohort. Furthermore, 53.0% early onset T2DM subjects developed microalbuminuria, a known risk factor for CVD. Although the macrovascular outcomes in our early onset population have not been obtained, the constellation of risk factors commonly encountered in this group suggests an extremely high tendency for cardiovascular disease. In consistent with previous studies, our data demonstrated that 50.7% early onset T2DM patients had developed atherosclerotic plaques in carotid artery at the mean age of 39.5 years. The evidence for increased cardiovascular morbidity in younger patients was confirmed by a large prospective cohort study of 4857 American Indian children who were followed up for 24 years [28]. In this study, obesity, glucose intolerance, and hypertension increased the risk of premature death by 130%, 73%, and 57%, respectively.

What makes things worse is that the patients with early onset T2DM had poor control of cardiovascular risk factors [8, 15]. Similar findings were observed in our study: 57.6% and 91.3% of patients with early onset T2DM did not achieve the recommended targets for blood pressure and lipids, respectively. Meanwhile, 45.2% of them were current smokers. Recent studies demonstrated that early onset T2DM subjects had poor compliance to cardioprotective medications [29]. Only 31% and 37% of this population insisted on statins and antihypertensive medications, respectively [30]. Consistently, our findings showed that only 6.3%, 8.0%, and 66.4% patients of the early onset DM group insisted on statins, aspirin, and antihypertensive medications, respectively (Supplementary Table 2). Therefore, increasing the awareness of controlling the risk factors and improving the compliance to medications on hypertension and dyslipidemia are crucial to modify these risk factors.

Our data demonstrated that young females with T2DM had a ~3-fold hazard of atherosclerotic plaques compared with male counterparts, in agreement with studies in non-Chinese populations [4]. A previous study showed that young women with T2DM had an increasing risk of developing MI than men (10.8-fold versus 3.9-fold) [4]. Diabetes may increase the detrimental effects of the other risk factors and alleviate the protective effect of estrogens. However, there is no evidence to date that normalization of glucose level in young women with T2DM may reduce the risk of CVD. For this reason, guidelines for such patients are aimed at keeping the other risk factors under strict control in order to significantly reduce their detrimental effects on CVD. However, studies demonstrated that women with early onset T2DM were less likely to be treated. Only 11.1% and 22.2% of female subjects received statins and antihypertensive medications, respectively, compared with 38.7% and 35.5% of male subjects with a similar age [31]. This gender disparity in cardioprotective treatment is of concern since it may compound the burden of excess risk for cardiovascular disease in women with diabetes. The elevated cardiovascular risk in women with early onset T2DM should be kept in the mind of health care professionals. And cardiovascular risk factors should be actively identified and tightly controlled in this population.

### 4.1. Limitations

Our study has several limitations. First, the study patients were not representative of the general patients with T2DM in Xinjiang, China. Patients with severe diabetes were more likely to seek care in tertiary medical center; therefore, the reported prevalence of diabetic complications in this study would be higher than in the general population with T2DM. Second, we fully recognize that distinguishing type 1 from type 2 diabetes is increasingly more challenging for clinicians since the incidence of T2DM is dramatically rising in young adults. Some of these young patients with T2DM present with diabetic ketoacidosis. As the body weight of the general increases, many people with type 1 diabetes are also obese. In order to reduce misclassification bias, we excluded 132 cases (7.6% of entire cases). By excluding these more severe cases, we may underestimate the full impact of T2DM in young adults. Third, though, we found early and late onset T2DM patients had different risk factors for macrovascular disease, but the sample number was limited, especially for early onset T2DM. As a cross-sectional designed study, it does not lead to any causative conclusions and more large prospective designed investigations are needed.

## 5. Conclusions

In summary, our data delineate that early onset T2DM patients in Xinjiang, China, had a poor glycemic control and a high burden of atherogenic risk factors. The prevalence of micro- and macrovascular complications was astonishingly high in this population. Moreover, young women with T2DM were more susceptible to cardiovascular complications. This population requires more aggressive management on all cardiovascular risk factors.

## Figures and Tables

**Table 1:** Clinical and biochemical characteristics of patients with early versus late onset of T2DM and control subjects matched for age and sex.

Variable	Early onset	Late onset	*P* value	Control subjects	*P* value^#^
	*n* = 219	*n* = 1036		*n* = 478	
Age (years)	39.5 ± 10.8	60.2 ± 8.3	<0.001	39.4 ± 7.1	0.103
Female gender	78 (35.6)	476 (45.)	0.005	182 (38.1)	0.556
Age of diabetes diagnosis (years)	34.3 ± 7.6	51.4 ± 7.2	<0.001	—	—
Duration of diabetes (years)	6.6 ± 8.0	8.8 ± 6.1	<0.001	—	—
Diabetic family history	138 (63.0)	499 (39.8)	<0.001	102 (21.3)	<0.001
BMI (kg/m^2^)	25.2 ± 4.9	26.1 ± 3.6	0.002	24.2 ± 2.8	0.009
Current cigarette smoking^∗^	99 (45.2)	381 (36.8)	0.012	213 (47.2)	0.680
Hypertension	112 (51.1)	582 (56.2)	0.330	153 (33.9)	<0.001
SBP (mmHg)	141.9 ± 25.1	137.4 ± 22.8	0.015	120.6 ± 18.0	<0.001
DBP (mmHg)	79.8 ± 14.7	77.1 ± 14.5	0.023	72.3 ± 12.7	<0.001
Fasting blood glucose (mmol/L)	10.3 ± 4.4	9.9 ± 4.6	0.680	4.9 ± 0.5	<0.001
2-h postprandial glucose (mmol/L)	15.7 ± 5.4	14.1 ± 5.2	0.535	5.7 ± 1.3	<0.001
HbA_1c_,% (mmol/mol)	9.1 ± 2.4 (76 ± 26)	8.3 ± 2.2 (67 ± 23)	0.039	5.2 ± 1.1 (33 ± 11)	<0.001
Required insulin therapy	160 (73.1)	608 (58.7)	<0.001	—	—
Triglyceride (mmol/L)	2.4 ± 1.6	2.2 ± 1.4	0.405	1.6 ± 0.9	<0.001
Total cholesterol (mmol/L)	4.1 ± 1.3	4.6 ± 1.5	0.952	3.7 ± 1.2	<0.001
HDL cholesterol (mmol/L)	1.1 ± 0.4	1.2 ± 0.4	0.397	1.2 ± 0.2	<0.001
LDL cholesterol (mmol/L)	2.6 ± 1.1	2.5 ± 1.0	0.500	2.1 ± 0.6	<0.001

Data are means ± SD or *n* (%). *P* values were derived from chi-square test or Student's *t*-test. BMI: body mass index; SBP/DBP: systolic/diastolic blood pressure; HbA_1c_: hemoglobin A1c; HDL cholesterol: high-density lipoprotein cholesterol; LDL cholesterol: low-density lipoprotein cholesterol. ^∗^Current cigarette smoking, 1 or more cigarettes per day. #*P* value, early onset DM versus control subjects matched with age and sex.

**Table 2:** Multivariate logistic regression analysis in patients with early onset T2DM (*n* = 219) in order to obtain odds ratios (OR) for macrovascular complications.

Variables	OR	95% CI	*P* value
Sex (male = 1)	3.22	1.53–6.78	0.002
Age (years)	1.05	1.02–1.08	0.001
SBP (mmHg)	1.04	1.02–1.07	0.036
2-h postprandial glucose (mmol/L)	1.01	1.00–1.03	<0.001

CI: confidence interval; SBP: systolic blood pressure.

**Table 3:** Multivariate logistic regression analysis in patients with late onset T2DM (*n* = 1039) in order to obtain odds ratios (OR) for macrovascular complications.

Variables	OR	95% CI	*P* value
Sex (male = 1)	0.25	0.17–0.37	<0.001
Age (years)	1.05	1.03–1.07	<0.001
DBP (mmHg)	1.01	1.00–1.03	0.043
2-h postprandial glucose (mmol/L)	1.04	1.01–1.07	0.002
LDL-CHO (mmol/L)	1.18	1.01–1.38	0.035

CI: confidence interval; DBP: diastolic blood pressure; LDL-CHO: low density lipoprotein cholesterol.

## Abbreviations

T2DM: Type 2 diabetes mellitus
CVD: Cardiovascular disease
PG: Plasma glucose
2-h PG: 2-hour postprandial glucose
HbA_1c_: Hemoglobin A1c
BP: Blood pressure
MI: Myocardial infarction.

## References

[1] Geiss L. S., Wang J., Cheng Y. J. (2014). Prevalence and incidence trends for diagnosed diabetes among adults aged 20 to 79 years, United States, 1980-2012. *JAMA*.

[2] Feltbower R. G., McKinney P. A., Campbell F. M., Stephenson C. R., Bodansky H. J. (2003). Type 2 and other forms of diabetes in 0-30 year olds: a hospital based study in Leeds, UK. *Archives of Disease in Childhood*.

[3] Song S. H., Hardisty C. A. (2009). Early onset type 2 diabetes mellitus: a harbinger for complications in later years--clinical observation from a secondary care cohort. *QJM*.

[4] Hillier T. A., Pedula K. L. (2003). Complications in young adults with early-onset type 2 diabetes: losing the relative protection of youth. *Diabetes Care*.

[5] Pavkov M. E., Bennett P. H., Knowler W. C., Krakoff J., Sievers M. L., Nelson R. G. (2006). Effect of youth-onset type 2 diabetes mellitus on incidence of end-stage renal disease and mortality in young and middle-aged Pima Indians. *JAMA*.

[6] Luk A. O. Y., Lau E. S. H., So W. Y. (2013). Prospective study on the incidences of cardiovascular-renal complications in Chinese patients with young-onset type 1 and type 2 diabetes. *Diabetes Care*.

[7] Pan X. R., Li G. W., Hu Y. H. (1997). Effects of diet and exercise in preventing NIDDM in people with impaired glucose tolerance. The Da Qing IGT and Diabetes Study. *Diabetes Care*.

[8] Gong H., Pa L., Wang K. (2015). Prevalence of diabetes and associated factors in the Uyghur and Han population in Xinjiang, China. *International Journal of Environmental Research and Public Health*.

[9] Huo X., Gao L., Guo L. (2016). Risk of non-fatal cardiovascular diseases in early-onset versus late-onset type 2 diabetes in China: a cross-sectional study. *The Lancet Diabetes and Endocrinology*.

[10] Alberti K. G. M. M., Zimmet P. Z. (1998). Definition, diagnosis and classification of diabetes mellitus and its complications. Part 1: diagnosis and classification of diabetes mellitus provisional report of a WHO consultation. *Diabetic Medicine*.

[11] Hogg R. J., Furth S., Lemley K. V. (2003). National Kidney Foundation’s kidney disease outcomes quality initiative clinical practice guidelines for chronic kidney disease in children and adolescents: evaluation, classification, and stratification. *Pediatrics*.

[12] Li M. Z., Su L., Liang B. Y. (2013). Trends in prevalence, awareness, treatment, and control of diabetes mellitus in mainland China from 1979 to 2012. *International Journal of Endocrinology*.

[13] Grinstein G., Muzumdar R., Aponte L., Vuguin P., Saenger P., DiMartino-Nardi J. (2003). Presentation and 5-year follow-up of type 2 diabetes mellitus in African-American and Caribbean-Hispanic adolescents. *Hormone Research in Paediatrics*.

[14] Xu Y., Wang L., He J. (2013). Prevalence and control of diabetes in Chinese adults. *JAMA*.

[15] Li L., Ji L., Guo X. (2015). Prevalence of microvascular diseases among tertiary care Chinese with early versus late onset of type 2 diabetes. *Journal of Diabetes and its Complications*.

[16] Yu H., Xie L. F., Chen K. (2016). Initiating characteristics of early-onset type 2 diabetes mellitus in Chinese patients. *Chinese Medical Journal*.

[17] van Dieren S., Beulens J. W. J., van der Schouw Y. T., Grobbee D. E., Neal B. (2010). The global burden of diabetes and its complications: an emerging pandemic. *European Journal of Preventive Cardiology*.

[18] Eppens M. C., Craig M. E., Cusumano J. (2006). Prevalence of diabetes complications in adolescents with type 2 compared with type 1 diabetes. *Diabetes Care*.

[19] TODAY Study Group (2013). Rapid rise in hypertension and nephropathy in youth with type 2 diabetes: the TODAY clinical trial. *Diabetes Care*.

[20] Pavkov M. E., Knowler W. C., Bennett P. H., Looker H. C., Krakoff J., Nelson R. G. (2006). Increasing incidence of proteinuria and declining incidence of end-stage renal disease in diabetic Pima Indians. *Kidney International*.

[21] Paisey R. B., Paisey R. M., Thomson M. P. (2009). Protection from clinical peripheral sensory neuropathy in Alström syndrome in contrast to early-onset type 2 diabetes. *Diabetes Care*.

[22] Stratton I. M., Adler A. I., Neil H. A. (2000). Association of glycaemia with macrovascular and microvascular complications of type 2 diabetes (UKPDS 35): prospective observational study. *BMJ*.

[23] UK Prospective Diabetes study group (1998). Tight blood pressure control and risk of macrovascular and microvascular complications in type 2 diabetes: UKPDS 38. *BMJ*.

[24] ADVANCE Collaborative Group, Patel A., MacMahon S. (2008). Intensive blood glucose control and vascular outcomes in patients with type 2 diabetes. *The New England Journal of Medicine*.

[25] Morrish N. J., Wang S. L., Stevens L. K., Fuller J. H., Keen H., WHO Multinational Study Group (2001). Mortality and causes of death in the WHO Multinational Study of Vascular Disease in Diabetes. *Diabetologia*.

[26] Turner R. C., Millns H., Holman R. R. (1997). Coronary heart disease and risk factors in NIDDM--experience from the United Kingdom Prospective Diabetes Study. *Diabetologia*.

[27] Rodriguez B. L., Fujimoto W. Y., Mayer-Davis E. J. (2006). Prevalence of cardiovascular disease risk factors in U.S. children and adolescents with diabetes: the SEARCH for diabetes in youth study. *Diabetes Care*.

[28] Franks P. W., Hanson R. L., Knowler W. C., Sievers M. L., Bennett P. H., Looker H. C. (2010). Childhood obesity, other cardiovascular risk factors, and premature death. *The New England Journal of Medicine*.

[29] Hatunic M., Burns N., Finucane F., Mannion C., Nolan J. J. (2016). Contrasting clinical and cardiovascular risk status between early and later onset type 2 diabetes. *Diabetes and Vascular Disease Research*.

[30] Yeung R. O., Zhang Y., Luk A. (2014). Metabolic profiles and treatment gaps in young-onset type 2 diabetes in Asia (the JADE programme): a cross-sectional study of a prospective cohort. *The Lancet Diabetes & Endocrinology*.

[31] Wilmot E. G., Davies M. J., Yates T., Benhalima K., Lawrence I. G., Khunti K. (2010). Type 2 diabetes in younger adults: the emerging UK epidemic. *Postgraduate Medical Journal*.

