# Supplementary material for: The Constellation of Macrovascular Risk Factors in Early Onset T2DM: A Cross-Sectional Study in Xinjiang Province, China
Source: J Diabetes Res. 2018 Apr 3;2018:3089317. doi: 10.1155/2018/3089317 (PMC5903340; doi:10.1155/2018/3089317)
Supplement: Supplementary Materials — Supplemental Material includes two tables and can be found with this article online. [file 3089317.f1.docx]

# Table 1 – The prevalence of macro-vascular complications in patients with early versus late onset of T2DM according to different estimate（n,%）

| Variable | Early onset DM | | Late onset DM | | *P* value |
| --- | --- | --- | --- | --- | --- |
|  | total n=219 | | total n=1036 | |  |
|  | n | positive | n | positive |  |
| **Coronary vascular disease** |  |  |  |  |  |
| Self report^1^ | 219 | 17(7.8) | 1036 | 218(21.0) | <0.001 |
| Electrocardiogram^2^ | 219 | 35(16.2) | 1036 | 279(26.9) | <0.001 |
| CT angiography^3^ | 21 | 18(85.7) | 183 | 153(83.0) | <0.001 |
| Coronary calcification ^4^ | 58 | 19(32.8) | 243 | 201(82.7) | 0.726 |
| Coronary angiography^5^ | 2 | 2(100.0) | 109 | 81(74.3) | 0.416 |
| **Stroke** |  |  |  |  |  |
| Interview^6^ | 219 | 1(0.4) | 1036 | 135(13.0) | <0.001 |
| Brain imaging with CT | 89 | 10(11.2) | 714 | 249(34.9) | <0.001 |
| Brain imaging with MRI | 62 | 20(32.3) | 547 | 224(41.0) | 0.186 |
| Brain angiography | 0 | 0 | 13 | 13(100.0) | - |
| **Peripheral vascular disease** |  |  |  |  |  |
| Interview^7^ | 219 | 1(0.4) | 1036 | 61(5.9) | <0.001 |
| Ankle-brachial index^8^ | 164 | 32(19.5) | 897 | 270(30.1) | 0.006 |
| Duplex ultrasound | 54 | 2(3.7) | 583 | 54(9.3) | 0.168 |
| MR imaging | 3 | 0 | 5 | 1(20.0) | 0.401 |
| CT angiography | 4 | 3(75.0) | 76 | 51(67.1) | 0.742 |
| Digital subtraction angiography | 0 | 0 | 89 | 89(100.0) | - |
| **Atherosclerotic plaques** |  |  |  |  |  |
| Carotid ultrasound^9^ | 219 | 111(50.7) | 1036 | 832(80.3) | <0.001 |

# Values are expressed as percentages (%) for categorical variables. *P* values were derived from chi-square test.

# ^1^ Coronary vascular disease Self report: positive: patient was reported a history of coronary heart disease. ^2^ Electrocardiogram: positive: ST-T-wave abnormalities or Q waves. ^3^ CT angiography: positive: atherosclerotic plaque was seen. ^4^ Coronary calcification: positive: coronary artery calcification score>400. ^5^ Coronary angiography: positive: stenosis of any coronary vessel >50%. ^6^ Stroke Self report: positive: the patient was reported a history of ischemic stroke, intracerebral hemorrhage or transient ischemic attacks. ^7^ Peripheral vascular disease Self report: positive: the patient was reported a history of foot ulcers or limb amputation. ^8^Ankle-brachial index: positive: ABI<0.9. ^9^ Atherosclerotic plaques: average carotid intima media thickness was calculated and a B-score was formed as follows: 0=no alteration, 1=wall thickness <1 mm, 2=plaque 1 to 2 mm, 3=plaque 2 to 3 mm, 4=plaque >3 mm, and 5=total occlusion of the lumen. A single B-score >2 on any measurement was defined as carotid atherosclerotic plaque.

# Table-2 Anti-hypertensive and statin therapies among patients with hypertension in early DM vs late onset DM vs control groups

| Variable | Early onset+ HTN | Late onset+ HTN | HTN without DM | *P* value |
| --- | --- | --- | --- | --- |
| Male gender (n, %) | 76(67.9) | 361(61.6) | 87(56.9) | *-* |
| Age (years) | 36.9±9.9 | 59.4±8.2 | 39.1±3.8 | <0.001 |
| SBP (mm Hg) | 151.4±20.6 | 155.5±18.9 | 138.5±14.4 | <0.001 |
| DBP (mm Hg) | 88.8±14.4 | 87.1±14.0 | 76.8±10.0 | <0.001 |
| HTN history (years) | 3.4±4.6 | 7.6±6.1 | 2.4±3.7 | <0.001 |
| **Anti-hypertensives therapies** |  |  |  |  |
| None (n, %) | 41（33.6） | 99（17.0） | 48（31.4） | <0.001 |
| CCBs (n, %) | 13（11.6） | 110（19.0） | 31（20.3） | 0.023 |
| ACE-Is or ARBs (n, %) | 24（21.4） | 140（24.1） | 17（11.1） | 0.003 |
| Beta blockers (n, %) | 2（1.8） | 8（1.4） | 10（6.5） | <0.001 |
| Diuretics (n, %) | 4（3.6） | 10（1.7） | 23（15.0） | <0.001 |
| Dual therapy (n, %) | 20（17.6） | 117（20.1） | 20（15.0） | 0.231 |
| 3 or more medications (n, %) | 8（7.1） | 98（16.8） | 4（2.6） | <0.001 |
| **Other therapies** |  |  |  |  |
| Statin (n, %) | 7(6.3) | 198(34.0) | 9(5.9) | <0.001 |
| Aspirin (n, %) | 9(8.0) | 204(35.1) | 12(10.5) | <0.001 |

Values are expressed as mean ± standard deviation or percentages (%) for categorical variables. *P* value refers to analysis of variance test for continuous measures or χ^2^ test for categorical variables among three groups. Abbreviations: HTN, hypertension; SBP/DBP, systolic/diastolic blood pressure; CCB, calcium channel blocker; ACE-I, angiotensin converting enzyme inhibitor; ARB, angiotensin receptor blocker.
